# Directly observed therapy and risk of unfavourable tuberculosis treatment outcomes among an international cohort of people living with HIV in low‐ and middle‐income countries

**DOI:** 10.1002/jia2.25423

**Published:** 2019-12-08

**Authors:** April C Pettit, Cathy A Jenkins, Meridith Blevins Peratikos, Marcel Yotebieng, Lameck Diero, Cuong D Do, Jeremy Ross, Valdilea G Veloso, Denise Hawerlander, Olivier Marcy, Bryan E Shepherd, Lukas Fenner, Timothy R Sterling, for the International Epidemiology Databases to Evaluate AIDS (IeDEA) Consortium

**Affiliations:** ^1^ Department of Medicine Vanderbilt University Medical Center Nashville TN USA; ^2^ Vanderbilt Tuberculosis Center Nashville TN USA; ^3^ Department of Biostatistics Vanderbilt University Medical Center Nashville TN USA; ^4^ College of Public Health The Ohio State University Columbus OH USA; ^5^ Academic Model Providing Access To Healthcare (AMPATH) Eldoret Kenya; ^6^ Bach Mai Hospital Hanoi Vietnam; ^7^ TREAT Asia/amfAR – The Foundation for AIDS Research Bangkok Thailand; ^8^ Instituto Nacional de Infectologia Evandro Chagas Fundação Oswaldo Cruz Rio de Janeiro RJ Brazil; ^9^ Centre Intégré de Recherches Biocliniques d'Abidjan CIRBA Abidjan Côte d'Ivoire; ^10^ Centre INSERM U1219 Bordeaux Population Health University of Bordeaux Bordeaux France; ^11^ Institute of Social and Preventive Medicine University of Bern Bern Switzerland

**Keywords:** tuberculosis, directly observed therapy, body mass index, human immunodeficiency virus infection, antiretroviral therapy

## Abstract

**Introduction:**

Identification of persons living with human immunodeficiency virus (HIV)‐associated tuberculosis (TB) at increased risk for unfavourable TB outcomes would inform efforts to improve such outcomes. We sought to identify factors associated with a decreased risk of unfavourable TB treatment outcomes among people living with HIV‐infection (PLHIV) in low‐ and middle‐income countries (LMIC), with a specific focus on directly observed therapy (DOT) compared with self‐administered therapy (SAT) during the continuation phase of anti‐TB therapy.

**Methods:**

We conducted a retrospective cohort study among adults diagnosed with HIV‐associated TB in Africa, Asia and the Americas from 2012 to 2013; data were collected from 2012 to 2016. Unfavourable TB treatment outcomes (death during TB treatment, and TB treatment failure or recurrence) were defined according to World Health Organization criteria. Receipt of DOT was obtained at the site level and defined as ≥5 days of DOT per week. The person administering DOT and treatment location varied by site. Lack of receipt of DOT was defined as SAT. Multivariable logistic regression estimated the adjusted odds of unfavourable TB treatment outcomes.

**Results:**

Among 1862 adults with HIV‐associated TB included, 252 (13.5%) had unfavourable TB outcomes (226 deaths, 26 recurrences/failures). Overall, 1825 (98%) received DOT in the intensive phase and 1617 (87%) received DOT in the continuation phase. DOT in the continuation phase was not significantly associated with unfavourable TB outcomes (aOR 1.43, 95% CI 0.86 to 2.38) compared to SAT. Body mass index (BMI) change during anti‐TB treatment (per 2 units increase, aOR 0.74, 95% CI 0.68 to 0.82) and CD4^+^ count at TB diagnosis (200 vs. 50  cells/µL, aOR 0.54, 95% CI 0.39 to 0.73) were both independently associated with decreased odds of unfavourable TB treatment outcomes.

**Conclusions:**

In this large, international cohort of people living with HIV‐associated TB in LMIC who received intensive phase DOT, DOT during the continuation phase of anti‐TB therapy was not associated with a decreased odds of unfavourable TB treatment outcomes compared to SAT. Randomized trials evaluating the effect of continuation‐phase DOT on TB outcomes among PLHIV are needed.

## 1 Introduction

Tuberculosis (TB) remains a serious public health problem, particularly among people living with human immunodeficiency virus (PLHIV). TB is the leading cause of death among PLHIV worldwide, and people with HIV‐associated TB have higher rates of other unfavourable TB treatment outcomes, including TB recurrence or TB treatment failure, that require re‐treatment. In 2016, TB treatment success rates were 82% overall but only 77% for HIV‐associated TB 1. TB re‐treatment, in turn, is associated with even higher rates of unfavourable TB treatment outcomes (including death, recurrence and failure) 2, 3, drug resistance 4, 5, and more complicated and costly drug regimens.

Standard therapy for patients without a prior history of TB consists of a 2‐month intensive phase including isoniazid, rifampicin, pyrazinamide and ethambutol (HRZE) followed by a 4‐month continuation phase including isoniazid and rifampicin (HR) 6. Suboptimal adherence has been associated with unfavourable TB treatment outcomes 7 and the World Health Organization's End TB Strategy emphasizes patient‐centred treatment supervision 8. Therefore, directly observed therapy (DOT) has been considered the standard of care for TB treatment, regardless of HIV status 9. However, two previous systematic reviews 10, 11 and one meta‐analysis 12 did not reveal an association between DOT and decreased risk of TB recurrence or failure compared to self‐administered therapy (SAT). Most studies included in these analyses either excluded HIV‐positive persons or only included participants with unknown HIV test results.

While DOT in the two‐month intensive phase is associated with a decreased risk of TB relapse 13, we are unaware of studies assessing the effect of DOT specifically during the longer continuation phase of therapy. DOT is often not provided in low‐ and middle‐income countries (LMIC) 14 as it is less feasible due to limited staffing, increased cost and transportation difficulties compared to higher income countries. The objective of this study was to identify factors associated with unfavourable TB treatment outcomes including mortality, TB treatment failure and recurrence among PLHIV in a large, international cohort collaboration. We specifically sought to determine if DOT during the continuation phase of therapy was associated with a decreased risk of unfavourable TB treatment outcomes compared to SAT.

## 2 Methods

### 2.1 Study population

**Figure 1:**
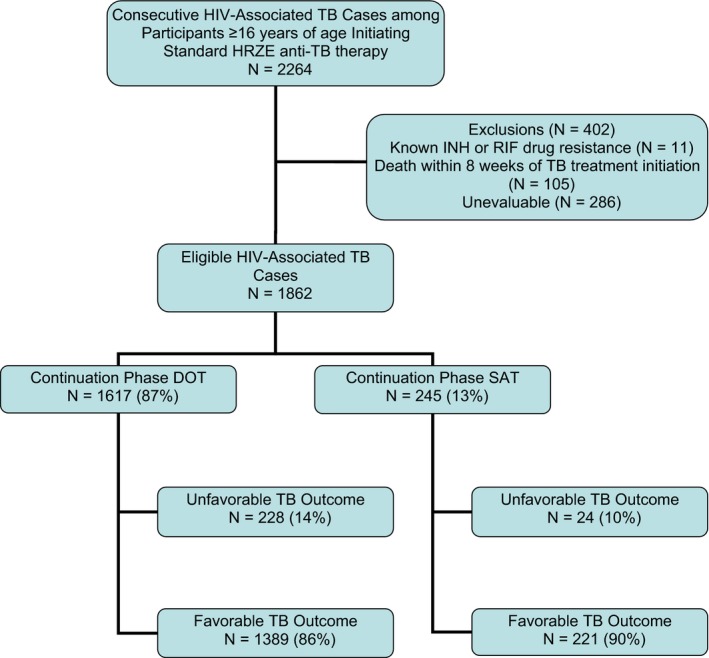
Flow diagram describing the study population. DOT, directly observed therapy; HIV, human immunodeficiency virus; HRZE, isoniazid/rifampicin/pyrazinamide/ethambutol; SAT, self‐administered therapy; TB, tuberculosis.

We conducted a retrospective cohort study among PLHIV aged ≥16 years from the International Epidemiology Studies to Evaluate AIDS (IeDEA) cohort collaboration (http://www.iedea.org/), a consortium of ART treatment programmes predominantly established in low‐ and middle‐income countries (LMIC). Sites were located in 18 countries and included a mix of healthcare facility types: referral hospitals, regional hospitals and primary care facilities. Consecutive participants were included if they were diagnosed with HIV‐associated TB between 1 January 2012 and 31 December 2013. Patients were followed until death, TB failure/recurrence or administrative censoring on 31 December 2014 in order to capture ≥12 months of follow‐up per individual. Patients who died within eight weeks of TB diagnosis were excluded as it may not have been possible to initiate ART within eight weeks, an important covariate of interest. Patients were excluded if they had documented resistance to isoniazid or rifampicin, were not initiated on the standard HRZE regimen for patients without a prior history of TB, or had unevaluable TB treatment outcomes (Figure 1).

### 2.2 Ethical considerations

Independent Ethics Committee (IEC) or Institutional Review Board (IRB) approval for this study was obtained by each of the local IeDEA sites as well as coordinating centres (see Data S1).

### 2.3 Data collection

A standardized case report form (CRF) designed for this study was used to collect data on patient demographics, vital status, laboratory data, TB treatment regimens and TB treatment outcomes. The CRF was developed in Research Electronic Data Capture (REDCap) and was available in English, French and Spanish 15. Local IeDEA site investigators completed a CRF for each TB case after medical record review from January 2012 to January 2016. Routine data audits for quality assurance were completed by the principal investigator (PI). Information on ART initiation dates were obtained from the electronic regional IeDEA data repositories.

### 2.4 Study definitions

TB case definitions and treatment outcomes were defined according to WHO definitions 16. Our primary outcome of interest was a composite unfavourable endpoint of death during TB treatment and TB treatment failure or recurrence, as both of the latter two endpoints would result in the need for TB re‐treatment. TB recurrence was defined as re‐initiation of therapy for a diagnosis of TB following a TB episode with an outcome of cure or treatment completion. TB cure was defined as a case of bacteriologically confirmed TB at the beginning of treatment with negative smears or cultures in the last month of treatment and on ≥1 previous occasion. TB treatment completion was defined as a case for whom treatment was completed, but without record of smear or culture results. TB treatment failure was defined as a TB case for whom sputum smear or culture was positive at five months or later during TB treatment. Unevaluable TB treatment outcomes included those lost to follow‐up or without an assigned TB treatment outcome.

Our primary exposure of interest was receipt of TB treatment by DOT during the entire continuation phase of therapy. DOT data were obtained at the site‐level and applied to each individual patient receiving care at the site. Receipt of DOT was defined as ≥5 days of DOT per week; <5 days of DOT was defined as SAT. DOT was defined according to local guidelines with respect to the person administering therapy (family member/friend vs. healthcare worker) and the location of treatment (home/community‐based vs. healthcare facility‐based).

Covariates identified for inclusion in multivariable models *a priori* included age at TB diagnosis, sex, baseline body mass index (BMI), change in BMI from the date of anti‐TB treatment start to the date of anti‐TB treatment stop, smear status, culture status, bacteriologic TB confirmation, site of disease (extrapulmonary, pulmonary/both/unknown), CD4^+^ count at TB diagnosis, IeDEA region, year of TB diagnosis and ART initiation timing in relation to TB diagnosis. Bacteriologically‐confirmed disease was defined as a positive acid fast bacillus (AFB) smear, culture, Xpert MTB/RIF or other nucleic acid amplification test (NAAT). Extrapulmonary TB (EPTB) was defined as a case of TB involving organs other than the lungs. Pulmonary TB (PTB) was defined as a case of TB involving the lung even when extrapulmonary disease was also present 1. The CD4^+^ lymphocyte count at TB diagnosis was defined as the nearest available value 180 days before or up to 30 days after the date of TB diagnosis.

ART was defined as ≥3 antiretroviral drugs including nucleoside reverse transcriptase inhibitors (NRTIs), protease inhibitors (PIs), non‐nucleoside reverse transcriptase inhibitors (NNRTIs), or integrase inhibitors. Early ART initiation was defined as initiation within eight weeks of TB diagnosis. This ART initiation timing cutoff was chosen to be consistent with the WHO guidelines recommending ART initiation within eight weeks of TB diagnosis dating back to 2009 6. TB diagnosis date was defined as the date of anti‐TB therapy initiation.

### 2.5 Statistical analysis

Categorical and continuous variables were compared using the chi‐squared and Wilcoxon rank sum tests respectively. The association of DOT with the composite outcome was assessed using unadjusted and adjusted logistic regression in order to estimate the odds of unfavourable TB outcomes compared to SAT. Continuous predictor variables were modelled using restricted cubic splines with three knots. Covariates with missing data (excluding missing data for the combined primary outcome) were imputed once using predictive mean matching 17. Subjects with missing outcome data were assumed to have been alive and event free until the end of follow‐up. All *p*‐values were two‐sided and considered statistically significant if <0.05. R version 3.4.2 (http://www.r-project.org) was used for analysis and analysis scripts are available online (http://biostat.mc.vanderbilt.edu/ArchivedAnalyses).

### 2.6 Sensitivity analyses

We identified *a priori* two important sensitivity analyses to perform. In the first, we explored the most conservative assumption that all persons with missing vital status had died.

In the second, only participants with baseline CD4^+^ counts <50 cells/µL who survived at least two weeks after TB diagnosis were included, and the ART initiation timing covariate cutoff was changed from eight weeks to two weeks. This sensitivity analysis was done to be consistent with results of the STRIDE study which demonstrated that ART initiation within two weeks of TB diagnosis for patients with HIV‐associated TB and a baseline CD4^+^ count <50 cells/µL was associated with a lower rate of AIDS defining illnesses and death 18.

For the second sensitivity analysis, inverse probability weighted propensity score methods 19 were utilized rather than standard logistic regression to avoid overfitting the models due to a low event rate. We adjusted for confounders by weighting observations using inverse probability weights (IPW); specifically, the inverse of the probability of the observed exposure level given the observed values of covariates (confounders). Weighting by this inverse probability created a pseudo‐population in which the covariates of interest were balanced. We performed this analysis for our primary exposure of interest (DOT) as well as our other covariates of interest.

Standardized mean difference (SMD) plots 20 were used to assess the appropriateness of the weights. Standardized difference in means was used to compare balance in measured variables between exposed and unexposed subjects. For continuous exposures, the groups were divided at the median to calculate SMDs; for >2 categories, the groups were divided by the most prevalent versus the rest to calculate SMDs. An SMD equal to 0.10 means that the difference in means is within 10% of the pooled standard deviation.

## 3 Results

**Figure 2:**
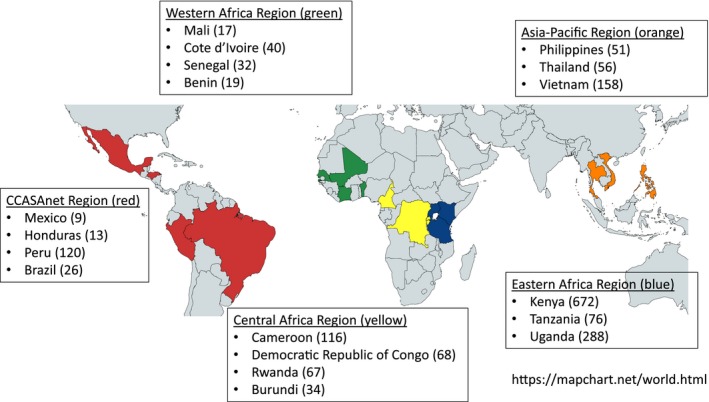
Adults with HIV‐associated TB included in the study by IeDEA region and country. Numbers in parentheses indicate the number of adults enrolled in each country.

There were 1862 adults aged 16 years or older diagnosed with HIV‐associated TB included in this analysis. Participants were from 18 countries in 5 IeDEA regions (Caribbean, Central, and South America network for HIV epidemiology (CCASAnet), Western Africa, Central Africa, Eastern Africa and the Asia‐Pacific) (Figure 2).

The clinical and demographic characteristics of the study population are in Table 1. Overall, 252 (13.5%) adults with HIV‐associated TB developed unfavourable TB outcomes. This included 226 deaths during TB treatment (12.1%) and 26 TB recurrences or treatment failures (1.4%). Among 1351 (72.6%) PLHIV not on ART who survived at least eight weeks after TB diagnosis, 477 (35%) were started on ART within eight weeks and 874 (65%) were started on ART >8 weeks after TB diagnosis or not started on ART before the end of follow‐up. PLHIV who had unfavourable TB treatment outcomes were more likely to be older at the time of TB diagnosis (38 vs. 36 years, *p* = 0.012) and have lower median CD4^+^ counts at the time of TB diagnosis (74 vs. 123 cells/µL, *p* < 0.001); they were less likely to gain weight over the course of TB treatment (0 vs. 2 kg, *p* < 0.001). There were also regional differences, with those who had unfavourable treatment outcomes being more likely to reside in the CCASAnet or Central and Eastern Africa regions (Table 1).

**Table 1:** Demographic and clinical characteristics of the study population

	Unfavourable (n = 252)	Favourable (n = 1610)	*p*‐value*
Age at TB diagnosis, median (IQR)	38 (32 to 45)	36 (30 to 43)	0.012
Female, n (%)	104 (41%)	669 (42%)	0.99
BMI at TB diagnosis (kg/m^2^), median (IQR)	18 (16 to 20)	19 (17 to 21)	0.013
Missing, n (%)	54 (21%)	312 (19%)	
BMI change from TB treatment start to stop date, median (IQR)	0 (−1 to 1)	+2 (1 to 3)	<0.001
Missing, n (%)	139 (55%)	621 (39%)	
AFB smear result, n (%)			
Positive	72 (29%)	516 (32%)	0.10
Negative	109 (43%)	736 (46%)	
Not performed	71 (28%)	357 (22%)	
Missing	0 (0%)	1 (<1%)	
AFB culture result, n (%)			
Positive	16 (6%)	96 (6%)	0.58
Negative	14 (6%)	118 (7%)	
Not performed	222 (88%)	1395 (87%)	
Missing	0 (0%)	1 (<1%)	
Bacteriologic Confirmation (smear, culture or NAAT positive), n (%)			
Yes	80 (32%)	596 (37%)	0.12
No	172 (68%)	1014 (63%)	
Site of disease, n (%)			
Pulmonary	176 (70%)	1140 (71%)	0.31
Extra‐pulmonary only	66 (26%)	374 (23%)	
Both	10 (4%)	82 (5%)	
Unknown	0 (0%)	14 (1%)	
Intensive phase DOT, n (%)			
Yes	251 (100%)	1574 (98%)	0.09
No	1 (<1%)	36 (2%)	
Continuation phase DOT, n (%)			
Yes	228 (90%)	1389 (86%)	0.08
No	24 (10%)	221 (14%)	
CD4^+^ count at TB diagnosis, median (IQR)	74 (26 to 197)	123 (45 to 262)	<0.001
Missing, n (%)	46 (18%)	250 (16%)	
ART status at TB diagnosis, n (%)			
No	174 (69%)	1177 (73%)	0.90
Yes	56 (22%)	366 (22%)	
Missing	22 (9%)	67 (4%)	
ART initiation after TB diagnosis (excluding those already on ART), n (%)^a^			
>8 weeks	105 (60%)	769 (65%)	0.23
8 weeks or less	69 (40%)	408 (35%)	
Region, n (%)			
Asia‐Pacific	14 (6%)	251 (16%)	<0.001
CCASAnet	39 (15%)	129 (8%)	
Central Africa	37 (15%)	248 (15%)	
Eastern Africa	151 (60%)	885 (55%)	
Western Africa	11 (4%)	97 (6%)	
Year of TB diagnosis, n (%)			
2012	140 (56%)	875 (54%)	0.77
2013	112 (44%)	735 (46%)	

Percentages are calculated for non‐missing data. AFB, acid fast bacilli; ART, antiretroviral therapy; BMI, body mass index; DOT, directly observed therapy; IQR, interquartile range; kg, kilogram; m, meter; NAAT, nucleic acid amplification test; TB, tuberculosis.

a The denominators for these percentages are the number of patients not on ART at TB diagnosis.

* *p*‐value is for the comparison of characteristics of persons with and without unfavourable outcomes.

The details regarding DOT at each site with respect to the number of days per week of DOT in the intensive phase, the number of days per week of DOT in the continuation phase, the person giving DOT, and the location of DOT are in Table 2. There were 37 (2%) PLHIV in care at one site who did not receive DOT during the intensive phase and 245 (13%) PLHIV in care at seven sites who did not receive DOT during the continuation phase of TB treatment. Only 13 (5%) of those categorized as not receiving DOT during the continuation phase of therapy (<5 days per week), received thrice weekly DOT; the remainder (95%) did not receive any DOT during the continuation phase of therapy.

**Table 2:** Directly observed therapy details by site

Site	Days per week IP	Days per week CP	Person giving DOT	Location of DOT
Benin	7	0	HCW	HCF
Brazil	5	5	HCW	Home or HCF
Burundi	7	7	Family, friend, or HCW	Home, community, or HCF
Cameroon	7	0	HCW	Healthcare facility
Cote d'Ivoire	7	7	Family or friend	Home or community
Honduras	6	3	Family, friend, or HCW	Home, community, or HCF
Kenya	7	7	Family, friend, or HCW	Home or HCF
Mali	7	7	Family, friend or HCW	Home, community or HCF
Mexico	5, 6, or 7	5	HCW	Home or HCF
Peru	5 or 6	5	HCW	HCF
Philippines	7	7	Family, friend, or HCW	Home, community, or HCF
Republique Democratique du Congo	6	0	HCW	HCF
Rwanda	7	7	HCW	Home, community, or HCF
Senegal	7	7	Family, friend, or HCW	Home, community, or HCF
Tanzania‐Kisesa	7	7	Family, friend, or HCW	Home or HCF
Tanzania‐Tumbi	7	0	HCW	HCF
Thailand‐HIV‐NAT	5, 6, or 7	0	Family, friend, or HCW	Community or HCF
Thailand‐Ramathibodi Hospital	0	0	No DOT	No DOT
Uganda	7	7	Family, friend, or HCW	Home, community, or HCF
Vietnam‐Bach Mai Hospital	7	7	Family, friend, or HCW	HCF during IP and home during CP
Vietnam‐NHTD	7	7	Family, friend, or HCW	Home, community, or HCF

CP, continuation phase; HCF, healthcare facility; HCW, healthcare worker; HIV‐NAT, The HIV Netherlands Australia Thailand Research Collaboration; IP, intensive phase; NHTD, National Hospital for Tropical Diseases.

In the adjusted models, continuation phase DOT was not significantly associated with a decreased odds of unfavourable TB treatment outcomes (aOR 1.43, 95% CI 0.86 to 2.38). Both a higher CD4^+^ count at TB diagnosis (200 vs. 50 cells/µL, aOR 0.54, 95% CI 0.39 to 0.73) and BMI increase during anti‐TB treatment (2 vs. 0 kg/mg^2^, aOR 0.74, 95% CI 0.68 to 0.82) were associated with a decreased odds of unfavourable TB treatment outcomes in the multivariable model. There was no association with the timing of ART initiation and TB treatment outcomes among those not already on ART at the time of TB diagnosis (Table 3).

**Table 3:** Factors associated with Unfavourable TB Outcomes (death, recurrence, failure)

	Adjusted odds ratio* (95% CI)
Age (years)	1.22 (0.99, 1.49)
Female sex	1.09 (0.81, 1.47)
**Baseline BMI (kg/m^2^)**	**0.61 (0.42, 0.88)**
**BMI change (2 kg/m^2^ increase vs. no change)**	**0.74 (0.68, 0.82)**
Smear positive	1.00 (0.42, 2.37)
Culture positive	1.28 (0.63, 2.61)
Bacteriologically confirmed	0.86 (0.36, 2.08)
Extrapulmonary versus pulmonary/both/unknown	1.18 (0.84, 1.66)
**CD4^+^ cell count (200 vs. 50 cells/µL)**	**0.54 (0.39, 0.73)**
ART status	
On ART at TB diagnosis versus ART initiation within eight weeks	1.08 (0.73, 1.58)
On ART at TB diagnosis versus ART after eight weeks or never started on ART	1.25 (0.87, 1.80)
Continuation phase DOT	1.43 (0.86, 2.38)

Bold value indicates statistical significant (*p* < 0.05).

ART, antiretroviral therapy; BMI, body mass index; DOT, directly observed therapy; TB, tuberculosis.

* The model was adjusted for all covariates in the table as well as year of TB diagnosis and IeDEA region.

In the first sensitivity analysis, 31 (1.7%) persons with missing vital status were assumed to have died and results were largely unchanged (see Table S1). In the second sensitivity analysis, 468 (25.1%) persons with CD4^+^ counts ≤50 cells/µL at TB diagnosis were included (Table 4). Of the 364 persons not on ART who survived at least two weeks after TB diagnosis, 49 (13%) were started on ART within two weeks of TB diagnosis and 315 (87%) were started on ART >2 weeks after TB diagnosis or not started on ART before the end of follow‐up. The adjusted model using IPW indicated a significant association between BMI change during anti‐TB treatment and a decreased odds of unfavourable TB treatment outcomes (aOR 0.60, 95% CI 0.49 to 0.73 for each 2 kg/m^2^ increase in BMI).

**Table 4:** Factors associated with unfavourable TB treatment outcomes adjusted using Inverse Probability Weighting and excluding those with CD4^+^ counts >50 cells/µL at TB diagnosis (n = 1394, 74.9%)

	IPW odds ratio* (95% CI)
Age (40 vs. 30 years)	1.04 (0.71, 1.52)
Female versus male	0.86 (0.52, 1.42)
**BMI change (2 kg/m^2^ increase vs. no change)**	**0.60 (0.49, 0.73)**
Smear positive	0.61 (0.36, 1.05)
Culture positive	0.99 (0.21, 4.74)
Bacteriologically confirmed	0.69 (0.41, 1.17)
Extrapulmonary versus pulmonary/both/unknown	1.13 (0.67, 1.91)
CD4^+^ cell count (30 vs. 10 cells/µL)	0.83 (0.57, 1.22)
ART status	
On ART at TB diagnosis versus ART initiation within two weeks	1.70 (0.73, 3.95)
On ART at TB diagnosis versus ART initiation after two weeks	1.12 (0.54, 2.32)
Continuation phase DOT	1.07 (0.33, 3.50)

Bold value indicates statistical significant (*p* < 0.05).

ART, antiretroviral therapy; BMI, body mass index; DOT, directly observed therapy; IPW, inverse probability weighting; TB, tuberculosis.

* Each summary is a separate model with IPW using all other factors. For change in BMI, BMI at treatment initiation was included in the inverse probability weighting model. In all models, IeDEA region and year of diagnosis were included in the inverse probability weighting model.

## 4 Discussion

In this study conducted within a large international HIV cohort collaboration, DOT during the continuation phase of TB treatment was not significantly associated with a decreased odds of unfavourable TB treatment outcomes (TB death, TB recurrence and TB treatment failure). Only 2% of this study population did not receive intensive phase DOT. It is possible that among a population with a very high frequency of DOT in the intensive phase, that DOT during the continuation phase does not affect TB treatment outcomes. There also could have been confounding by indication – that persons at increased risk of unfavourable TB treatment outcomes preferentially received continuation phase DOT. A previous randomized trial conducted in Pakistan did not reveal an association between DOT and TB outcomes when given only during the intensive phase, compared to SAT throughout the entire course of anti‐TB treatment 21.

Several previous studies have attempted to evaluate the impact of DOT on TB treatment outcomes but did not focus on the population of PLHIV, did not focus on DOT in the continuation phase of anti‐TB therapy, and did not use a composite endpoint of unfavourable TB treatment outcomes (death, failure and recurrence). In three prospective studies (two conducted in Thailand and one in Taiwan), DOT was shown to be associated with a decreased risk of TB treatment default 22, 23, 24 and increased rates of TB treatment cure 23, 24. One of these studies also showed a trend towards a decreased risk of TB treatment failure with DOT compared to SAT 23. DOT has not been shown to be associated with decreased risk of TB relapse 25, 26, 27, although these studies were also limited by low numbers of relapse events.

The rate of unfavourable TB treatment outcomes in this population of persons with HIV‐associated TB who survived at least eight weeks was 13.5%; the TB mortality rate was 12.1% and the TB recurrence or treatment failure rate was 1.4%. Globally in 2017, the WHO estimated that the TB case fatality rate among people living with HIV was 32.6%. Moreover it is estimated that 23% of TB notifications among PLHIV had unfavourable treatment outcomes 1. It is possible that the rates of unfavourable outcomes in this population are lower than global estimates because this population was enrolled from a cohort of PLHIV who were in care in an ART programme. It is also likely that the exclusion of persons who died within the first eight weeks of TB treatment (n = 106, 32%) and/or the possibility that some deaths were missed contributed to the lower TB mortality rate in this study population.

The majority of patients not on ART at the time of TB diagnosis (65%) in our study who survived at least eight weeks after TB diagnosis, were not started on ART within eight weeks. Among those with CD4^+^ counts <50 cells/µL at TB diagnosis, even a higher proportion who survived at least two weeks after TB diagnosis (87%) were not started on ART within two weeks. This finding is consistent with that of a previous study in South Africa, which found that only 51.1% initiated ART within eight weeks of TB diagnosis. These data suggest that barriers to expedited ART initiation exist and that improved integration of TB and ART services may lead to improvements in ART initiation delays 28.

We did not find an association with the timing of ART initiation and TB treatment outcomes among those not already on ART at the time of TB diagnosis. Several clinical trials have shown that earlier ART initiation in relation to TB diagnosis is associated with improved survival 18, 29, 30; however, there are several significant differences between those studies and our study. Only one of these trials, the STRIDE study, allowed enrolment of persons with smear‐negative TB as our study did. The STRIDE study used a primary endpoint of AIDS‐defining illness and death. Of their 116 endpoints, 63 (54%) were due to AIDS‐defining illnesses and 53 (46%) were due to death 18. In contrast, our study used a primary endpoint of TB recurrence or treatment failure and death. Of our 252 endpoints, 26 (10%) were due to TB recurrence or treatment failure and 226 (90%) were due to death. It is possible that these differences in eligibility criteria and primary outcome definitions led to our differing findings with respect to the timing of ART initiation.

An increase in BMI during TB treatment was associated with a decreased risk of TB recurrence or treatment failure. This finding is consistent with the findings from Tuberculosis Trials Consortium Study 22 which found that participants who were underweight at enrolment and had a <5% weight gain over the intensive phase of anti‐TB treatment had nearly twice the risk of relapse compared to those who gained more than 5% 31. It is unclear if the association between lack of weight gain and TB recurrence or treatment failure is due to a poor response to anti‐TB treatment stunting the rate of weight gain, or if the presence of adequate weight gain is required in order for patients to respond appropriately to anti‐TB therapy. Regardless, the WHO recommends nutritional evaluation, counselling and support as part of TB care 32.

The main limitation of this study is that we did not have detailed data at the individual patient level with respect to adherence either in the setting of DOT or SAT. Moreover we did not have individual level data on the person administering DOT (family member/friend vs. healthcare worker) or the location of DOT (home/community‐based vs. healthcare facility‐based). However, a previous meta‐analysis found no difference in TB treatment outcomes between community‐observed versus family‐observed and between home‐observed versus healthcare facility‐observed DOT 10. Another limitation was that the number of TB recurrences and treatment failures was low, which decreased the power of the study. While we did not collect information regarding whether a smear and/or culture at months 5 or later during TB treatment was performed or not, we do know that 22% of cases did not have a smear performed at any time during TB treatment and 86% of cases did not have a culture performed at any time during TB treatment. So, misclassification of TB treatment failure as another outcome is possible due a lack of smear and/or culture for TB diagnosis. Additionally, it is possible that some of the cases that transferred care to another facility or defaulted may have actually had an unfavourable TB outcome including death, recurrence or failure.

Moreover it is possible that some recurrent cases were not identified during the 12‐month follow‐up. Two studies reporting combined results from TB treatment trials suggest that 12 months of follow‐up is sufficient in order to capture most TB recurrences 33, 34. However, it may take longer to identify recurrent TB cases in programmatic settings in which follow‐up is less active compared to clinical trials. Additionally, it is possible that some patients moved out of the local IeDEA site catchment area which would also contribute to under‐ascertainment of recurrent TB cases.

Another limitation was the lack of data on medical comorbidities known to be associated with unfavourable TB outcomes. One important example, includes diabetes mellitus (DM). In a recent systematic review, patients with TB and comorbid DM had a higher odds of death and relapse 35. Future studies evaluating the impact of factors associated with unfavourable TB outcomes should include DM and other important medical comorbidities.

## 5 Conclusions

In this large, multi‐centre, international study among individuals with HIV‐associated TB who received intensive phase DOT, we found that DOT given during the continuation phase of TB treatment was not associated with a decreased odds of unfavourable TB treatment outcomes. A higher CD4^+^ count at TB diagnosis was independently associated with a decreased odds of unfavourable TB treatment outcomes, highlighting the need for early HIV testing and treatment. Increasing BMI during anti‐TB treatment was also associated with a decreased odds of unfavourable TB treatment outcomes supporting the importance of nutritional evaluation and support during anti‐TB therapy. Randomized trials evaluating the impact of DOT during the continuation phase of therapy on TB outcomes among PLHIV are needed.

## Competing interest

All authors report no conflicts of interest related to this work.

## Authors' contributions

ACP, MBP, LF and TRS contributed to study conception and design. MY, LD, CDD, JR, VGV, DH and OM contributed to acquisition of data. ACP, CAJ, MBP, BES, LF and TRS contributed to analysis and interpretation of data. ACP contributed to drafting of the manuscript. ACP, CAJ, MBP, MY, LD, CDD, JR, VGV, DH, OM, BES, LF and TRS contributed to critical revision of the manuscript for important intellectual content. All authors have reviewed and approved the final, submitted version of the manuscript.

## Supporting information


**Data S1.** Ethics Statement.

**Data S2.** Membership of the International Epidemiologic Databases to Evaluate AIDS (IeDEA) participating programs.

**Table S1.** Factors associated with Unfavorable TB treatment outcomes when those with missing vital status (n = 31, 1.7%) were assumed to have died
