# Supplementary material for: Directly observed therapy and risk of unfavourable tuberculosis treatment outcomes among an international cohort of people living with HIV in low‐ and middle‐income countries
Source: J Int AIDS Soc. 2019 Dec 8;22(12):e25423. doi: 10.1002/jia2.25423 (PMC6900483; doi:10.1002/jia2.25423)
Supplement: Supplementary file 1 — Data S1. Ethics Statement. [file JIA2-22-e25423-s001.docx]

**Ethics Statement**

Independent Ethics Committee (IEC) or Institutional Review Board (IRB) approval for this study was obtained by each of the local IeDEA sites as well as from the Vanderbilt University IRB and included: Indiana University IRB, Comite de Etica do Instituto Nacional de Infectologia Evandro Chagas-Fiocruz (Brazil), Comité de Éticas en Investigación Biomédica (CEIB) of the Unidad de Investigación Científica (UIC) (Honduras), Comité de Ética en Investigación del Instituto Nacional de Ciencias Médicas y Nutrición, Salvador Zubirán (Mexico), Comité Institucional de Ética para Humanos (CIEH)  and  Comité Institucional de Ética en Investigación del Hospital Cayetano Heredia (CIEI) (Peru), Comité national d’éthique pour la recherche en santé (CNERS) (Benin), Comité National d'Ethique et de la Recherche (Côte d'Ivoire), Comité National d'Ethique pour la santé et les Sciences de la vie (Mali), Comité National d'Ethique pour la Recherche en Santé (Senegal), Moi University/Moi Teaching and Referral Hospital Institutional Research and Ethics Review Committee (Kenya), The United Republic of Tanzania National Institute for Medical Research Coordinating Committee (Tanzania-Tumbi and Kisesa), Mbarara University of Science and Technology Institutional Research Ethics Committee (Uganda), Comité National d’Ethique (Burundi), Cameroon National Ethical Committee of Research for Human Health (Cameroon), Comite D’ethique of the University of Kinshasa School of Public Health (République Démocratique du Congo), Rwanda National Ethics Committee (Rwanda), Research Institute for Tropical Medicine Department of Health (Philippines), Committee on Human Rights Related to Research Involving Human Subjects Faculty of Medicine Ramathibodi Hospital, Mahidol University (Thailand), Institutional Review Board Faculty of Medicine, Chulalongkorn University (Thailand, HIV- NAT), Ministry of Health, Hanoi School of Public Health Institutional Review Board (Bach Mai Hospital, Vietnam)*, The Ethical Review Board for Biomedical Research of National Hospital of Tropical Diseases (Vietnam). Written informed consent was waived by all committees except for the Research Institute for Tropical Medicine Department of Health (Philippines), Institutional Review Board Faculty of Medicine, Chulalongkorn University (Thailand, HIV-NAT), and Ministry of Health, Hanoi School of Public Health Institutional Review Board (Bach Mai Hospital, Vietnam). These three sites enrolled only participants >18 years of age, so a separate consent procedure for minors was not required.
