# Supplementary material for: Directly observed therapy and risk of unfavourable tuberculosis treatment outcomes among an international cohort of people living with HIV in low‐ and middle‐income countries
Source: J Int AIDS Soc. 2019 Dec 8;22(12):e25423. doi: 10.1002/jia2.25423 (PMC6900483; doi:10.1002/jia2.25423)
Supplement: Supplementary file 2 — Data S2. Membership of the International Epidemiologic Databases to Evaluate AIDS (IeDEA) participating programs. [file JIA2-22-e25423-s003.docx]

**Membership of the International Epidemiologic Databases to Evaluate AIDS (IeDEA) participating programs**

**IeDEA Caribbean, Central, and South America (CCASAnet), www.ccasanet.org:**

**Instituto Nacional de Infectologia-Fiocruz, Brazil:** Beatriz Grinsztejn, Valdilea Veloso, Paula Luz, Raquel de Boni, Sandra Cardoso Wagner, Ruth Friedman, Ronaldo Moreira.

**Instituto Hondureño de Seguridad Social, Honduras:** Denis Padgett.

**Instituto Nacional de Ciencias Médicas y Nutrición Salvador Zubirán, Mexico**: Juan Sierra Madero, Brenda Crabtree Ramirez, Paco Belaunzaran, Yanink Caro Vega.

**Instituto de Medicina Tropical Alexander von Humboldt, Peru**: Eduardo Gotuzzo, Fernando Mejia, Gabriela Carriquiry.

**Vanderbilt University Medical Center, USA:** Catherine C McGowan, Bryan E Shepherd, Timothy Sterling, Karu Jayathilake, Anna K Person, Peter F Rebeiro, Mark Giganti, Jessica Castilho, Stephany N Duda, Fernanda Maruri, Hilary Vansell.

**IeDEA Asia Pacific,** [**www.amfar.org/treatasia**](http://www.amfar.org/treatasia)**:**

R Ditangco, MK Pasayan and ML Mationg, Research Institute for Tropical Medicine, Muntinlupa City, Philippines; A Avihingsanon, S Gatechompol, P Phanuphak and C Phadungphon, HIV-NAT/Thai Red Cross AIDS Research Centre, Bangkok, Thailand; S Kiertiburanakul, A Phuphuakrat, L Chumla and N Sanmeema, Faculty of Medicine Ramathibodi Hospital, Mahidol University, Bangkok, Thailand; KV Nguyen, HV Bui, DTH Nguyen and DT Nguyen, National Hospital for Tropical Diseases, Hanoi, Vietnam; CD Do, AV Ngo and LT Nguyen, Bach Mai Hospital, Hanoi, Vietnam; AH Sohn, JL Ross and B Petersen, TREAT Asia, amfAR - The Foundation for AIDS Research, Bangkok, Thailand;MG Law, A Jiamsakul, R Bijker and D Rupasinghe, The Kirby Institute, UNSW Sydney, NSW, Australia.

**East Africa IeDEA,** **www.iedea-ea.org/joomla:**

Diero L, Ayaya S, Sang E, MOI University, AMPATH Plus, Eldoret, Kenya; John Ssali, Mathew Ssemakadde, Masaka Regional Referral Hospital, Masaka, Uganda; Kapella Ngonyani, Jerome Lwali, Tumbi Regional Hospital, Pwani, Tanzania; Mark Urassa, Denna Michael, Richard Machemba, National Institute for Medical Research (NIMR), Kisesa HDSS, Mwanza, Tanzania; Kara Wools-Kaloustian, Constantin Yiannoutsos, Rachel Vreeman, Beverly Musick, Indiana University School of Medicine, Indiana University, Indianapolis, IN, USA; Batya Elul, Columbia University, New York City, NY, USA; Jennifer Syvertsen, Ohio State University, Columbus, OH, USA; Rami Kantor, Brown University/Miriam Hospital, Providence, RI, USA; Jeffrey Martin, Megan Wenger, Craig Cohen, Jayne Kulzer, University of California, San Francisco, CA, USA; Paula Braitstein, University of Toronto, Toronto, Canada

**West Africa IeDEA,** **www.mereva.net/iedea:**

***Benin, Cotonou****:* Djimon Marcel Zannou, Carin Ahouada, Jocelyn Akakpo, Christelle Ahomadegbé, Jules Bashi, Alice Gougounon-Houéto, Angèle Azon-Kouanou, Fabien Houngbé, Jean Sehonou (CNHU Hubert Maga). ***Côte d’Ivoire, Abidjan:*** Henri Chenal, Denise Hawerlander, Franck Soppi (CIRBA); ***Mali, Bamako:*** Hamar Alassane Traore, Daouda Minta, Tidiani Cissé, Mamadou Dembelé, Mohammed Doumbia, Mahamadou Fomba, Assétou Soukho Kaya, Abdoulaye M Traoré, Hamady Traoré, Amadou Abathina Toure (CH Point G). ***Senegal, Dakar:*** Moussa Seydi, Papa Salif Sow, Bernard Diop, Noël Magloire Manga, Judicael Malick Tine^§^, Coumba Cissé Bassabi (SMIT, CHU Fann). **Executive Committee:** François Dabis (Principal Investigator, Bordeaux, France), Emmanuel Bissagnene (Co-Principal Investigator, Abidjan, Côte d’Ivoire), Elise Arrivé (Bordeaux, France), Patrick Coffie (Abidjan, Côte d’Ivoire), Didier Ekouevi (Abidjan, Côte d’Ivoire), Antoine Jaquet (Bordeaux, France), Valériane Leroy (Bordeaux, France), Charlotte Lewden (Bordeaux, France), Annie J Sasco (Bordeaux, France). **Operational and Statistical Team:** Dieudonné Amani (Abidjan, Côte d’Ivoire), Jean-Claude Azani (Abidjan, Côte d’Ivoire), Eric Balestre (Bordeaux, France), Serge Bessekon (Abidjan, Côte d’Ivoire), Franck Bohossou (Abidjan, Côte d’Ivoire), Camille Gilbert (Bordeaux, France), Sophie Karcher (Bordeaux, France), Jules Mahan Gonsan (Abidjan, Côte d’Ivoire), Jérôme Le Carrou (Bordeaux, France), Séverin Lenaud (Abidjan, Côte d’Ivoire), Célestin Nchot (Abidjan, Côte d’Ivoire), Karen Malateste (Bordeaux, France), Amon Roseamonde Yao (Abidjan, Côte d’Ivoire), Bertine Siloué (Abidjan, Côte d’Ivoire). **Administrative Team:** Gwenaelle Clouet (Bordeaux, France), Madikona Dosso (Abidjan, Côte d’Ivoire), Alexandra Doring (Bordeaux, France), Adrienne Kouakou (Abidjan, Côte d’Ivoire), Elodie Rabourdin (Bordeaux, France), Jean Rivenc (Pessac, France). **Consultants/ Working Groups:** Xavier Anglaret (Bordeaux, France), Boubacar Ba (Bamako, Mali), Jean Bosco Essanin (Abidjan), Andrea Ciaranello (Boston, USA), Sébastien Datté (Abidjan, Côte d’Ivoire), Sophie Desmonde (Bordeaux, France), Jean-Serge Elvis Diby (Abidjan, Côte d’Ivoire), Geoffrey S.Gottlieb (Seattle, USA), Apollinaire Gninlgninrin Horo (Abidjan, Côte d’Ivoire), Serge N'zoré Kangah (Abidjan, Côte d’Ivoire), Denis Malvy (Bordeaux, France), David Meless (Abidjan, Côte d’Ivoire) , Aida Mounkaila-Harouna (Bordeaux, France), Camille Ndondoki (Bordeaux, France), Caroline Shiboski (San Francisco USA), Boris Tchounga (Abidjan, Côte d’Ivoire), Rodolphe Thiébaut (Bordeaux, France), Gilles Wandeler (Dakar, Senegal). **Coordinating Centre:** ISPED, Univ Bordeaux Segalen, Bordeaux, France **Regional Office:** PAC-CI, Abidjan, Côte d’Ivoire **Methodologic Support**: MEREVA, Bordeaux, France

**Central Africa IeDEA,** [**www.iedeaca.org**](http://www.iedeaca.org)**:**

Jean Claude Dusingize and Eugene Mutimura, Andre Gitembagara (Women’s Equity in Access to Care and Treatment, Kigali Rwanda); Kathryn Anastos (Albert Einstein College of Medicine and Montefiore Medical Center, New York, USA); Judy Tatwangire, Izimukwiye Izabelle, (Rwanda Military Hospital, Kigali, Rwanda); Theodore Niyongabo, Christelle Twizere (Centre hospitalo-Universitaire de Kamenge, Bujumbura, Burundi); Evelyne Baramperanye, (Centre National de Reference en matiere du VIH, Bujumbura ,Burundi); Andrew Edmonds, Marcel Yotebieng (Kalembelembe Pediatric hospital, Kinshasa, DRC); Innocent Azinyue, Liliane Ayangma, (Military Hospital of Yaoundé).
