# Supplementary material for: Directly observed therapy and risk of unfavourable tuberculosis treatment outcomes among an international cohort of people living with HIV in low‐ and middle‐income countries
Source: J Int AIDS Soc. 2019 Dec 8;22(12):e25423. doi: 10.1002/jia2.25423 (PMC6900483; doi:10.1002/jia2.25423)
Supplement: Supplementary file 3 — Table S1. Factors associated with Unfavorable TB treatment outcomes when those with missing vital status (n = 31, 1.7%) were assumed to have died [file JIA2-22-e25423-s002.doc]

**Factors associated with Unfavorable TB treatment outcomes when those with missing vital status (n=31, 1.7%) were assumed to have died**

|  | IPW Odds Ratio* (95% CI) |
| --- | --- |
| Age (40 vs. 30 years) | 1.04 (0.71, 1.52) |
| Female vs. Male | 0.86 (0.52, 1.42) |
| **BMI change (2 kg/m^2^ increase vs. no change)** | **0.60 (0.49, 0.73)** |
| Smear positive | 0.61 (0.36, 1.05) |
| Culture positive | 0.99 (0.21, 4.74) |
| Bacteriologically Confirmed | 0.69 (0.41, 1.17) |
| Extrapulmonary vs. pulmonary/both/unknown | 1.13 (0.67, 1.91) |
| CD4+ cell count (30 vs 10 cells/mL^3^) | 0.83 (0.57, 1.22) |
| ART status |  |
| On ART at TB Diagnosis vs. ART initiation within 2 weeks | 1.70 (0.73, 3.95) |
| On ART at TB Diagnosis vs. ART initiation after 2 weeks | 1.12 (0.54, 2.32) |
| Continuation Phase DOT | 1.07 (0.33, 3.50) |
|  |  |
|  |  |
|  | IPW Odds Ratio* (95% CI) |

Abbreviations: TB, tuberculosis; BMI, body mass index; kg, kilogram; m, meter; ART, antiretroviral therapy; DOT, directly observed therapy

*The model was adjusted for all covariates in the Table as well as year of TB diagnosis and IeDEA region.
